# Organization and implementation of an oral cholera vaccination campaign in an endemic urban setting in Dhaka, Bangladesh

**DOI:** 10.1080/16549716.2019.1574544

**Published:** 2019-02-15

**Authors:** Iqbal Ansary Khan, Ashraful Islam Khan, Anisur Rahman, Shah Alam Siddique, Md. Taufiqul Islam, Md. Amirul Islam Bhuiyan, Atique Iqbal Chowdhury, Nirod Chandra Saha, Prasanta Kumar Biswas, Amit Saha, Fahima Chowdhury, John D. Clemens, Firdausi Qadri

**Affiliations:** a Medical Social Science, Institute of Epidemiology Disease Control and Research (IEDCR), Dhaka, Bangladesh; b Infectious Diseases Division, International Centre for Diarrhoeal Disease Research (ICDDR), Dhaka, Bangladesh

**Keywords:** Cholera, organization, implementation, feasible, vaccination, endemic, urban, Bangladesh

## Abstract

Bangladesh has historically been cholera endemic, with seasonal cholera outbreaks occurring each year. In collaboration with the government of Bangladesh, the Infectious Diseases Division, International Centre for Diarrhoeal Disease Research, Bangladesh (icddr,b) initiated operational research to test strategies to reach the high-risk urban population with an affordable oral cholera vaccine (OCV) “ShancholTM” and examine its effectiveness in reducing diarrhea due to cholera. Here we report a sub-analysis focusing on the organization, implementation and effectiveness of different oral cholera vaccine delivery strategies in the endemic urban setting in Bangladesh. We described how the vaccination program was planned, prepared and implemented using different strategies to deliver oral cholera vaccine to a high-risk urban population in Dhaka, Bangladesh based on administrative data and observations made during the program. The objective of this study is to evaluate the organization, implementation and effectiveness of different oral cholera vaccine delivery strategies in the endemic urban setting in Bangladesh. OCV administration by trained local volunteers through outreach sites and mop-up activities yielded high coverage of 82% and 72% of 172,754 targeted individuals for the first and second dose respectively, using national Expanded Program on Immunization (EPI) campaign mechanisms without disrupting routine immunization activities. The cost of delivery was low. Safety and cold chain requirements were adequately managed. The adopted strategies were technically and programmatically feasible. Current evidence on implementation strategies in different settings together with available OCV stockpiles should encourage at-risk countries to use OCV along with other preventive and control measures.

## Background

Over the last few years, safe and effective use of the oral cholera vaccine (OCV) in emergencies, disasters, and even endemic settings has prompted its use as an adjunct to accepted public health tools to combat cholera [1–9]. Furthermore, widespread use of the OCV can have indirect ‘herd immunity’ effects [10]. The World Health Organization (WHO) is now strongly considering OCV as a control measure for endemic cholera in addition to other established control measures [11–13]. Accordingly, the WHO has created a global stockpile of OCV for cholera control [14]. The Global Alliance for Vaccine board recently approved a contribution of 20 million OCV doses over the next five years to this stockpile to increase access in outbreak situations and endemic settings. This will enable broader use of the OCV in settings where it can provide a valuable complement to traditional efforts to improve water and sanitation [15,16].

Bangladesh has suffered from various pandemics over the last two centuries [17–20]. The exact magnitude and distribution of disease in Bangladesh are unknown due to a lack of adequate and equitable diagnostic facilities and effective disease surveillance systems. Cholera-related mortality has, however, decreased due to heightened awareness of the use of oral rehydration salts, better accessibility to healthcare facilities, and proper case management. Nevertheless, as an endemic country, Bangladesh still bears the brunt of cholera throughout the year [21–26].

On this backdrop, a large feasibility study titled ‘Introduction of Cholera Vaccine in Bangladesh (ICVB)’ was initiated with the objectives of (ⅰ) developing and testing strategies to reach populations most at risk of cholera in endemic areas with OCV and (ⅰⅰ) examining its effectiveness in reducing diarrhea due to cholera. ‘Shanchol^TM^’, a low-cost OCV was delivered between 17 February and 16 April 2011 with logistic support from the EPI of the Directorate General of Health Services (DGHS). Reports on vaccine coverage, program cost, and vaccine effectiveness are presented elsewhere [9,27–29]. This paper is a descriptive report of organization and implementation of oral cholera vaccination campaign in a high-risk endemic urban population in Dhaka, Bangladesh.

## Materials and Methods

### Study area and population

**Figure 1.**
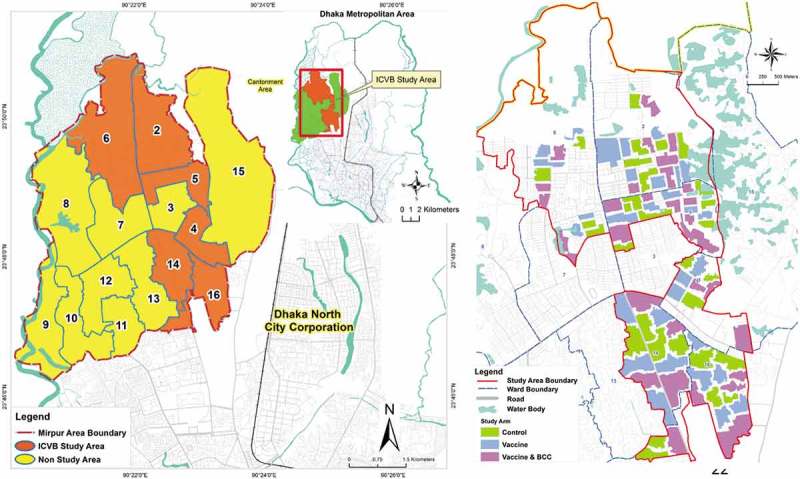
(a) ICVB study area in the six wards of Mirpur, Dhaka, Bangladesh. (b) The geographic clusters of the three arms of the ICVB project.

Bangladesh is a densely populated developing country in South East Asia, where a large population is always at high risk of cholera transmission due to inadequate access to safe water, poor sanitation and hygiene practices [30,31]. To test the vaccination strategies, six densely populated wards (ward 2, 4, 5, 6, 14, and 16) in Mirpur in Dhaka metropolis were selected as the study area (Figure 1(a)) based on the hospital records of 2–6 cholera cases per 1,000 diarrheal hospitalizations from those wards. A baseline census was conducted using maps developed with geographical information system (GIS). Around 317,000 high-risk individuals were identified living in the study area] along with a low-risk population [9]. For the effectiveness analysis, the study area was divided into 90 clusters (Figure 1(b)), each containing around 2,700 high-risk individuals with a 30-meter buffer area around them. These clusters were then randomly assigned to the three study arms (each containing 30 clusters): vaccination only, vaccination and behavior change intervention and a non-intervention arm (control).

**Figure 2.**
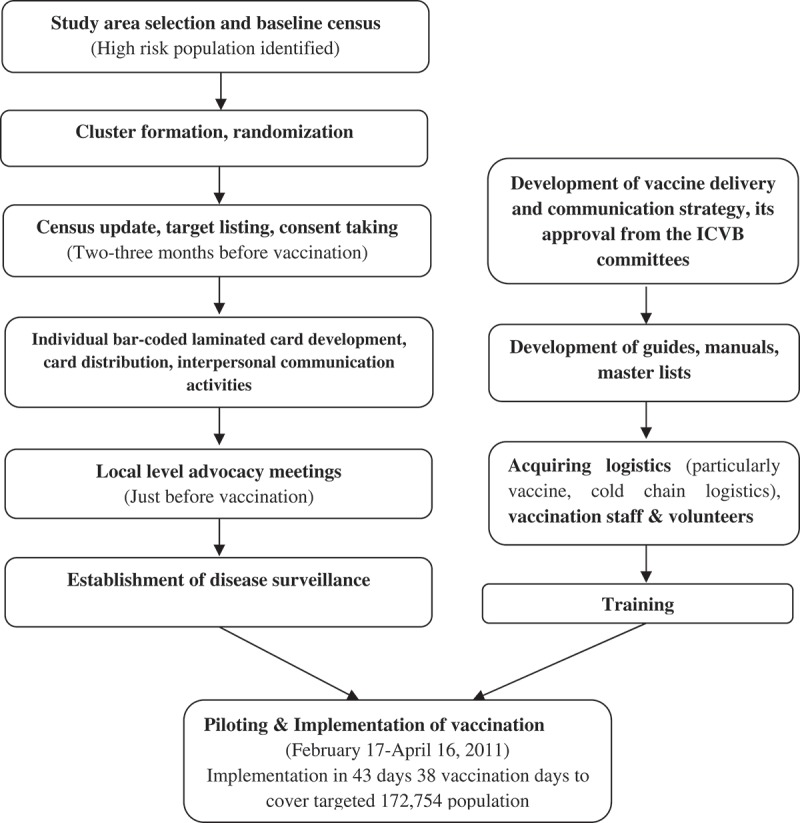
Activity flow for the mass vaccination program.

Just before vaccination, a census update identified 172,754 target individuals in the 60 vaccine clusters excluding children under one year of age and pregnant women. All individuals were issued a bar-coded card with a unique personal identification number and approached for written informed consent for vaccination. The program implementation and activity flow for the mass vaccination program is shown in Figure 2.

### Social mobilization

The planning and implementation, steering, and advisory committees were formed with the study investigators and representatives from the government, NGOs, the WHO and UNICEF to oversee program activities. A press release was issued to the media detailing the ICVB study in Mirpur. Several committee meetings were organized to refine program implementation. Before vaccination, several advocacy meetings were arranged with all stakeholders including City Corporation officials, ward councilors, and community representatives. Just before vaccination, field workers and volunteers visited each targeted household, distributed the ICVB card, and conveyed messages about the cholera vaccination program. On vaccination days, field workers and volunteers reminded the communities to attend the vaccination site with the ICVB cards. Cell phone messaging was also used to remind them of the vaccination. On the vaccination days, banners were visible at the outreach vaccination sites and on the vaccine-carrying pickup trucks.

### Vaccination strategy and other preparatory activities

This vaccination program used existing EPI cold chain logistics. A fixed outreach site vaccine delivery strategy was adopted to deliver two doses of OCV in two rounds at a minimum 14-day interval. Provision was made for mop-up activities after the second round for those who missed the second dose. The 60 vaccine clusters were grouped into five cycles. In each cycle of three days, 12 clusters were planned for vaccination, after which the vaccination teams moved to the next cycle. Therefore, 15 vaccination days were required in each round to cover sixty clusters. In the first round, a pilot program was planned in the three clusters of the first cycle to test whether the strategies were implementable. The adopted vaccination strategy had adequate flexibility to improve quality, accessibility, and coverage.

**Figure 3.**
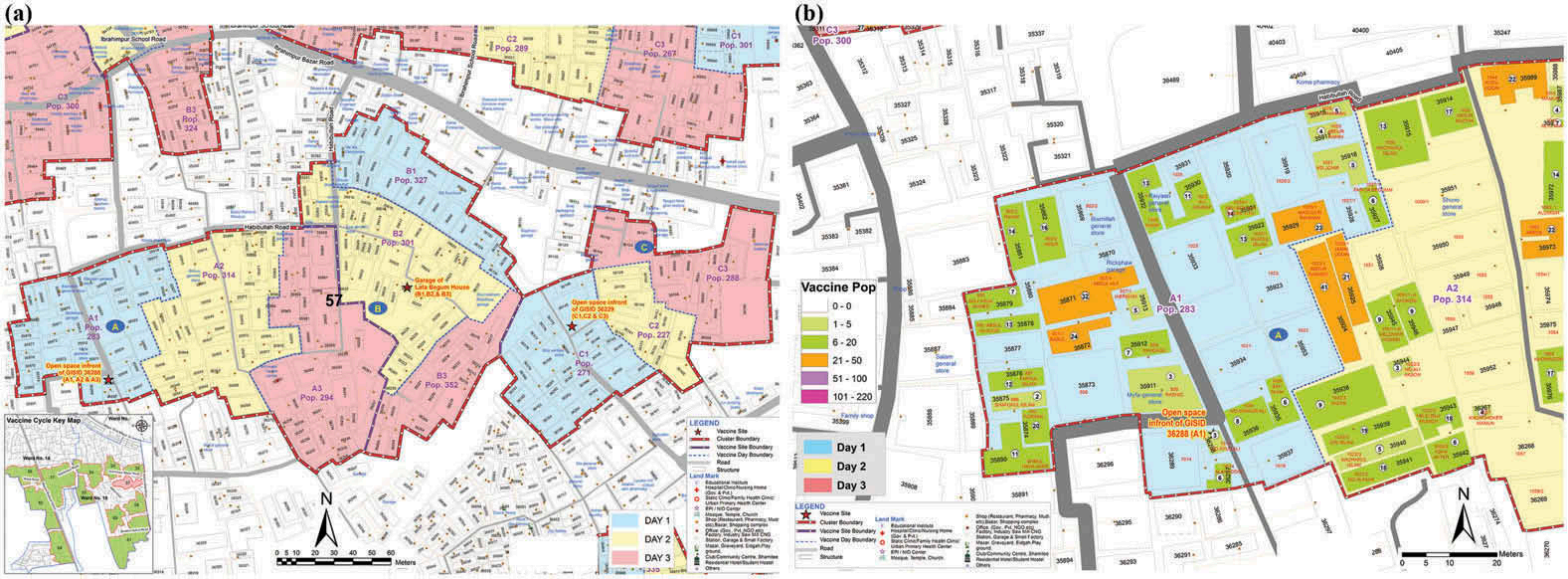
(a) Vaccination site (A, B, C) area in a cluster. (b) Target households and population in vaccination day-wise map.

To help efficient vaccine delivery, each cluster area was divided into three fixed outreach vaccination sites (A, B, and C). Thus, a 100 and 80 primary sites were identified. Each site was selected in consultation with the community at a convenient place for around 900 high-risk people. Places were chosen that were (i) available, (ii) known to the community, (iii) easily accessible, and (iv) previously used for immunization or similar activities. Several alternative sites in a few clusters were also selected for better accessibility. Target populations at each site were again divided over the three days and invited by the community mobilizers on the specified days of vaccination for each cluster. Cluster maps showing the three vaccination site areas, (Figure 3(a)) and targeted households and populations for vaccination on each day (Figure 3(b)) were prepared. The volunteers effectively used these maps to mobilize people for vaccination.

To record vaccination, session reports with master lists for each site in the vaccine clusters were prepared, which included participant lists with names, addresses, and other identifiers, including personal identification numbers, structure or building identification numbers, mobile numbers, cluster-specific vaccination serial numbers and date of each round of vaccination. The format had space to record information regarding the participant’s eligibility for vaccination, i.e. whether the individual consented for vaccination, was aged over one, was not pregnant, and was not severely ill. Information on age and consent (if previously given) were pre-printed on the form. The bar-coded ICVB cards were prepared with all individual identifiers. For easy identification, the cards were marked with a single dot for vaccine arm, two dots for vaccine and BCC arm, and no dot for the control arm. Any vaccine-eligible cardholders had the opportunity to get the vaccine on any day at any of the ongoing sites if they missed their scheduled date.

Implementation manuals were developed for training and program implementation. Along with the existing census staff, community volunteers were recruited and trained to assist vaccine promotion and delivery. Emphasis was given to organizing a vaccination session; controlling and mobilizing the crowd; identifying the target population using maps and master lists; maintenance of the vaccine cold chain; vaccine delivery; record keeping; reporting; supervision; taking informed consent for vaccination; vaccine transportation; waste management; and adverse events following immunization (AEFI) and their management. During three days of piloting, nine teams were deployed to nine sites in the three clusters, and each day nine other teams shadowed the vaccinating teams. Thus, all the teams were given hands-on training in the field before the scheduled vaccine delivery in other clusters.

Passive AEFI surveillance was established at the vaccination sites and in 13 health facilities in and around the study area. An AEFI was defined as any adverse event reported by the vaccinee with an onset within 14 days of receipt of any vaccine dose. During mobilization and also at the vaccination sites, participants were told to report any adverse event at the sites or at the designated health facilities.

### Vaccine and other logistics management

Shanchol^TM^ was chosen as the OCV due to its ease of administration in mass vaccination campaigns and relatively low cost. Considering 100% coverage with 10% wastage, 350,000 vaccine doses were procured with approval from the Directorate of Drug Administration of Bangladesh. Based on available cold space at EPI, vaccines were shipped in two lots by air, the first arriving just before piloting and the second before the second round of vaccination. At all stages, vaccines were kept between 2°C and 8°C. On each vaccination day at around 6 am, the required vaccines along with 10% possible wastage for each site were transported from the EPI cold room in 20 liter cold boxes. A foam pad was placed on top of frozen icepacks at the bottom, and conditioned icepacks were placed against the walls of the box. Vaccines in their paper cartons in a polythene bag were then placed inside the box along with a dial thermometer, with conditioned icepacks placed on top. About 20–24 icepacks were used in each cold box. On each vaccination day, 36 cold boxes containing the required vaccines were sent to vaccination sites, with one reserve box kept at the field office along with two icepack-containing boxes. According to distribution plan and route maps, all cold boxes reached their intended destinations in three pickups by 7 am. At the vaccination site, the vaccinators took out 50–60 vaccine vials in individual paper jackets from the cold box with four conditioned icepacks and kept them in the carrier. When delivered, the carrier was replenished with vaccines from the cold box. Icepacks, if melted, were also replaced from the cold box. On each vaccination day, the temperature of the cold boxes was recorded at three specific time points using the dial thermometer kept inside.

EPI provided the required carriers, cold boxes, dial thermometers, and icepacks in addition to vaccine storage space in two walk-in cooler and freezer rooms. Except for the vaccines, other logistics such as chairs, tables, water jars, glasses, etc. were sent to the vaccination sites on the day before the start of vaccination in each cluster. Three small pickup trucks were used to transport the vaccines, waste, and for other logistics. Second-line supervisors used five more vehicles during the vaccination period.

### Micro-planning, session management, record keeping, and data management

Forty-five days before vaccination, three sites were selected in each cluster, each to vaccinate around 900 participants over three days. ICVB cards, master lists, and micro-plans for each cluster were prepared with site addresses, target populations, vaccination dates, and site-specific resources.

Based on available cold chain logistics, 36 vaccination teams worked in the 12 clusters of a cycle on each vaccination day. Considering around 300 vaccinations per team per day, each team had two vaccinators and six other staff with specific responsibilities (record keeping/marking, crowd control, community mobilization, attending vaccinees). Several vaccinators and volunteers were kept in reserve to support overcrowded sites or for deployment to additional sites. Volunteers circulated the date and time for vaccination to the target households and motivated them to attend for vaccination on the scheduled dates. After completion of 12 clusters in a cycle, the teams moved to the next 12 clusters for three days and, in this way, all 60 clusters were covered in two rounds.

**Figure 4.**
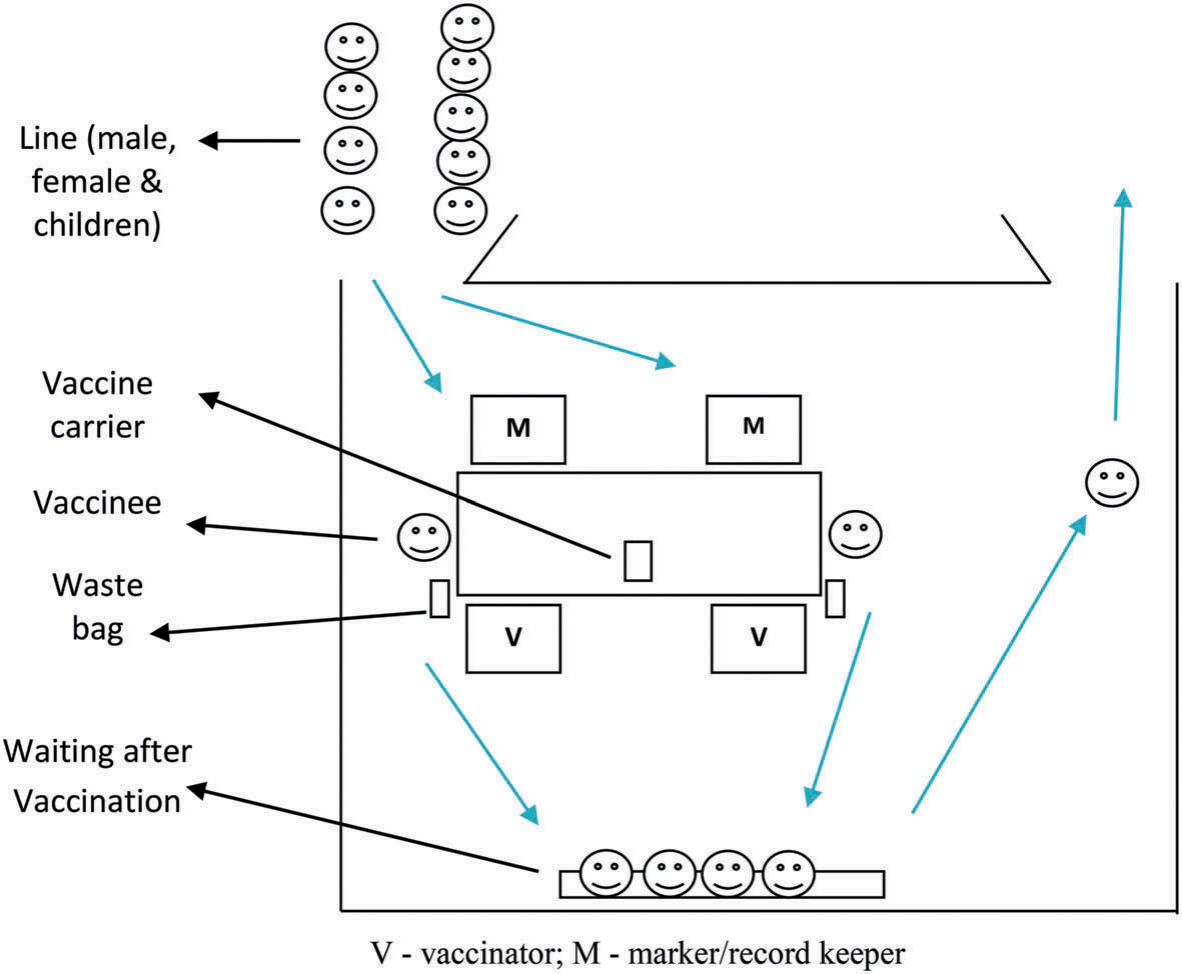
Team set up in the vaccination center.

On the morning of vaccination days, the teams set up the sites (Figure 4), informed and mobilized the target population, and delivered the vaccines. Before vaccination, children’s ages were verified with guardians. The pregnancy status of married women was verified by asking their menstrual history. If not consented earlier, consent was obtained at the vaccination site. In both rounds, vaccination dates were recorded in the session reports and on the ICVB cards. If a vaccine recipient spitted or vomited out the vaccine, s/he was revaccinated and recorded as such. A few blank session report sheets were kept at each site for recording the vaccination of participants from other sites or new participants in the clusters. The completed master list and session reports were effectively used to track down missing participants and drop-outs and mobilize them for vaccination in each round. At the end of the third day’s vaccination at a site, the supervisors submitted the completed session reports for data entry, which was performed simultaneously. After completion of two rounds of fixed outreach sessions, second dose omissions in each cluster were identified, listed, and plotted in cluster maps for mop-up. Six mop-up teams, each comprising one vaccinator with a vaccine carrier and a record keeper, were deployed in each cluster, and 12 clusters were covered each day. Mop-up vaccination records were transferred to the session reports and were also entered into the computers.

In total, 78 vaccinators, 220 volunteers, 12 first-line supervisors, and around 20 second-line supervisors from the ICVB project, Dhaka City Corporation, and EPI were also involved with vaccination program implementation. Additionally, six physicians were involved with AEFI surveillance in the hospitals and 15 people with three small pickups were responsible for monitoring the packing, distribution, and transportation of vaccines, logistics, and waste management.

According to the three sites per cluster strategy, 180 sites were required. However, in clusters with disperse populations, where available, more than three sites were selected for better accessibility. As such, 19 clusters had four sites and two clusters had five sites. The sites were established in open spaces on the roadside (74), households (57), institutional facilities like schools, kindergartens, hospitals, and clinics (37), and social gathering places such as clubs, shops, and mosques (35). These fixed outreach vaccination sites were used in two rounds for one to three consecutive days to deliver the two doses.

First round vaccination in 60 clusters including piloting for delivering the first dose continued until 13 March, 2011. No mop-up by home visit was planned after the first round, but the first dose omissions (those who missed their scheduled time) were given the opportunity to get the vaccine from any site during the second round (for delivering the second dose) between 15 March and 1 April, 2011. To provide yet another opportunity to complete two doses for those who received the first dose, a five-day mop-up campaign for the five cycles was organized on 7, 8, 9, 15, and 16 April, 2011 by visiting their households. A fixed site was kept open until 16 April 2011 at the ICVB study field office for any omissions.

## Results

Of the total 141,877 first doses, 129,799 (91.48%) and 11,991 (8.45%) were delivered in the first and second round, respectively; 27 (0.019%) first doses were delivered through the fixed office site and, during mop-up, 60 (0.04%) unintended first doses were also delivered (Table 1).

**Table 1.** Delivered vaccines through different strategies.

Vaccination Strategies		Dose 1 n (%)	Dose 2 n (%)	Total dose delivered
Fixed outreach site	1st round (Feb 17-Mar 13)	129,799 (91.48)	-	129,799
	2nd round (Mar 15- Apr 1)	11,991 (8.45)	110,373 (89.23)	122,364
Fixed office site (Mar 5 – Apr 16)		27 (0.019)	300 (0.24)	327
Mop up (7,8,9,15 and 16 Apr)		60 (0.04)	13,029 (10.53)	13,089
Total		141,877*	123,694**	265,571***

*Includes incomplete 6 first doses and **3 second doses; ***wasted doses are not included.

About 110,373 (89.23%), 13,029 (10.53%), and 300 (0.24%) second doses were delivered through second-round fixed outreach, mop-up, and the office site, respectively, resulting in 123,694 second doses in total.

The overall coverage was 82% and 72% for dose 1 and dose 2, respectively. Out of the 141,877 first dose and 123,694 second dose recipients, 84% and 63% received the respective doses on the scheduled dates. On average, each team with two vaccinators delivered 230 doses per day during the first round and 227 doses during the second round.

One thousand two hundred of the census population did not consent to take the vaccine and 1,107 consented first-dose recipients refused to take the second dose. Acutely ill participants (280 during the first dose and 171 during the second dose) were not dosed. About 1,906 pregnant women were not given their respective doses and 46,316 individuals were absent/migrated out during the vaccination program, about one-third (16,601) of whom took one dose but were not available to complete the two dose schedule (Table 2). 18,183 (13%) first-dose recipients did not attend for the second dose. Among these dropouts, most (37%) were 18–39 years of age (Table 3).

**Table 2.** Reasons for non-vaccination.

	<1yr age	Consent is not given/refusal	Pregnant	Suffering from acute illness	Absent/migrated out	Death before vaccination program	Incomplete dose	Total
Dose 1	2373	1200	1620	280	29,715	1	6	35,195
Dose 2	-	1107	286	171	16,601	-	3	18,178
Total	2373	2307	1906	451	46,316	1	9	53,373

**Table 3.** Distribution of different age groups of the dropouts.

Age groups	Frequency	Percentage
Age <1	8	0.0
Age 1–9	2362	13.0
Age 10–17	2665	14.7
Age 18–29	6887	37.9
Age 30–39	3107	17.1
Age 40–49	1713	9.4
Age 50 and above	1441	7.9
Total	18183	100.0

## Discussion

We reached 72% of the population with complete two dose. Our drop-out rate from first to second dose was 10%. Dropout was more among the young and middle-aged population. Adult male vaccination coverage was comparatively poor [9], particularly in adult workers in their twenties. It’s noteworthy that, in our vaccine effectiveness analysis the coverage shown was 65% [27]. The reason behind this coverage variation is because, population who had missed or not covered during the baseline census were included in the effectiveness analysis as zero time population (date of first dose for vaccine recipients).

To avoid conflict with the National Immunization Day vaccine campaigns, we delayed our program by one month to obtain support from immunization-related stakeholders, particularly the EPI for cold space and logistics. Due to this change, our vaccination schedule coincided with upazila and municipality elections and the national census, resulting in the movement of a large number of study participants who could not be vaccinated. Seasonality, low disease incidence period, avoiding other major community activities, and political issues must be taken into consideration when planning such programs.

Community participation in this program was notable, as reflected by community involvement in the program and vaccine coverage. Except for a few roadside vaccination sites, all sites were provided by the community and at all sites they provided space for overnight safekeeping of logistics.

In this study, the rate of vaccine delivery was low compared to that observed in the Sudanese refugee camp in Uganda (200 vaccinations by three operators in one hour) [3]. Even though this was a mass vaccination program, as a study requirement, consent was taken from each participant, non-eligible participants were screened out, and these efforts were time consuming and required extra hours, thereby impacting on the vaccination rate. In addition to the usual 10 am to 12 noon slot, vaccinations were efficiently delivered in the early morning (when people were on their way to work) and also during the middle of the day (when they returned home for lunch).

A considerable number of individuals did not consent to participate in the vaccination program as they were unavailable at the time of target listing or due to lack of awareness about cholera or this new vaccination program. However, during the vaccination program, a proportion of non-consented individuals attended the vaccination sites, consented, and received the vaccine. The shared experience of vaccination with neighbors and peers was instrumental in this phenomenon. Pregnant women were not given OCV due to safety concerns, although current evidence on the safety of the vaccine in pregnancy [32] suggest that the OCV will benefit this most vulnerable group in the future. The taste and flavor of the vaccine made many children and adolescent female participants vomit, which played a role in refusals during the first round and even more in the second round.

Mass vaccination with assistance from the mass media is known to yield better compliance. Here we avoided mass media communication due to the cluster randomized design of the intervention, mixed abodes of eligible and non-eligible populations within the same cluster, and the population living in buffer areas. There were several positive responses in the national and international media during the vaccination program. Nevertheless, the avoidance of mass media was exploited by one local newspaper and their negative reporting was responsible for the spread of rumors and a substantial number of refusals, particularly during the second round. Mass media campaign with interpersonal communication by field workers and institutional delivery, particularly in industry, factories and educational facilities, could improve compliance and minimize refusal and absence of the younger and middle-aged people.

For better accessibility, the vaccination sites were placed in the cluster communities and remained open for three days at each site. As we proceeded with vaccinating one cluster after another, ongoing monitoring revealed that there were many non-attendees in each cluster. Mop-up is effective but is laborious, costly and its management is complex. Furthermore, the availability of Mop-up diminishes individual responsibility and discourages attendance at outreach sites, which disrupts established routine practices. Fearing less attendance in the second round, we did not include mop-up after the first round but kept it after the second round. We retained the opportunity for non-attendees to get the first dose during the second round. This strategy helped 11,991 (8%) participants to get their first dose. The mop-up activities after the second round gave us the opportunity to improve two-dose coverage and to verify vaccination data records submitted during the regular program. Approximately 11% (13,029) of the delivered second doses were through mop-up activities, showing the effectiveness of this strategy.

To encourage participation, we used weekends and holidays for vaccination. We kept the door open for eligible newcomers in the clusters during the first round for the first dose. However, in the second round, we did not include any newcomers for vaccination.

The cold boxes, ice packs, and vaccine carriers were the reserves kept at EPI headquarters. Their use in the OCV program did not compromise any regular EPI activities. The card used had a tremendous impact on the program. First, it conveyed important messages about diarrhea, its management, and the program. It reminded participants about the two vaccine doses. It also acted as a link between the participant and the database in master list and helped volunteers to identify the site-specific appropriate participant for vaccination. Vaccine wastage was very low (1.2%), and reported adverse events were only 0.04%, the main symptoms being diarrhea and vomiting [9].

A major cost of any vaccination program is the vaccine itself and the cost related to human resources. In this study, costs for delivering the complete two doses to a person was US$ 0.70, and a single dose was US$0.33 excluding the cost for vaccines and salary support [9]. Shanchol^TM^ is still not affordable to low-income countries. Increased demand, more production, competition in the market, and local production in endemic countries may help to reduce prices and increase affordability. Recent evidences on the stability, safety and level of protective immunity at higher temperatures will reduce the cold chain requirement for these OCVs, thus making them less costly and more easily deliverable [33].

Due to cholera endemicity and the large population in Bangladesh, it is necessary to identify cholera high-risk areas/populations by establishing surveillance systems or using the existing reported data on diarrheal diseases. Resource wise, it is difficult to target the entire population at once, instead targeted vaccination in cholera high-risk areas/populations in a phase wise manner along with other control measures may be an effective and feasible approach for Bangladesh.

The immunization infrastructure in Bangladesh is relatively strong and stretches even to the remotest communities. In this study the existing cold room capacities of EPI were used effectively for cholera vaccines along with other EPI antigens. The strategies used for vaccine packing during transport and delivery adequately maintained the cold chain. Additional human resources for vaccine delivery for supplemental immunization activities are readily available from the community [9,34]. By using the existing cold chain system, government human resources and community volunteers, the overall cost for vaccine delivery will be low. OCV coverage could be further improved by using government machineries with proper media communication, facility-based extended service hours, institutional vaccine delivery, and an acceptable vaccine taste and odor.

## Conclusions

This is one of the largest vaccination programs using a two-dose OCV targeting a high-risk urban population in an endemic setting. The adopted strategies for OCV administration were technically and programmatically feasible to reach the target population. Coverage was satisfactory by using existing EPI infrastructure without disrupting routine immunization activities. Cold chain was well maintained without additional investment. The experiences from a large-scale vaccination campaign as demonstrated by this study could serve to inform and encourage cholera high-risk countries to use OCV along with other preventive measures.

## Paper context

OCV is an important public health tool to control cholera. This operational research was conducted to test strategies to reach high-risk urban population with an affordable oral cholera vaccine. This is one of the largest vaccination programs using a two dose oral cholera vaccine targeting a high-risk population in an endemic urban setting. This study shows that mass oral cholera vaccination of high-risk individuals in the endemic setting is technically and operationally feasible.
